# High-frequency turbidity by sensors as a proxy for total phosphorus: implications of sampling strategies on the water framework directive classification

**DOI:** 10.1038/s41598-026-44177-1

**Published:** 2026-03-12

**Authors:** Eva Skarbøvik, Anastasija Isidorova, Maria Kämäri, Pasi Valkama, Sofie G. M. van’t Veen, Emma E. Lannergård, Jens Fölster, Brian Kronvang

**Affiliations:** 1 Division of Environment and Natural Resources, Norwegian Institute of Bioeconomy Research (NIBIO), P.O. Box 115, Ås, 1431 Norway; 2 Finnish Environment Institute (Syke), Marine and freshwater solutions, Latokartanonkaari, 11, Helsinki, 00790 Finland; 3 Department of Ecoscience, Aarhus University, C.F. Møllers Allé, Aarhus C, Denmark and Envidan A/S, Vejlsøvej, 23, Silkeborg, 8600 Denmark; 4 Department of Aquatic Sciences and Assessment, Swedish University of Agricultural Sciences, PO Box 7050, Uppsala, 750 07 Sweden; 5 Department of Ecoscience, Aarhus University, C.F. Møllers Allé 3, Aarhus C, 8000 Denmark

**Keywords:** Stream, Turbidity, Phosphorus, Uncertainty, Sampling frequency, Climate sciences, Environmental sciences, Hydrology

## Abstract

Mean concentrations of pollutants and nutrients are frequently used in the classification of water bodies under the European Commission Water Framework Directive. Turbidity can be used as a proxy for several particle-associated substances. We used three years of sensor turbidity data in each of 10 Nordic streams to assess the uncertainty in the annual arithmetic mean concentration of turbidity when sampling weekly, fortnightly, or monthly. On average, weekly sampling produced an uncertainty of 17±9% for all streams, whereas monthly sampling increased this to 40±19%. We then developed a multiple linear model that shows how the uncertainty in annual mean turbidity increases with decreasing sampling frequency, decreasing catchment size, and increasing proportion of agricultural land in the catchment. As an example, a river of 800 km^2^ catchment area and 20% agricultural land will need 29 samples a year to give a mean annual turbidity within 10% uncertainty, but a stream of 10 km^2^ catchment area and 80% agricultural land will need 95 samples to achieve the same uncertainty. Turbidity was a suitable proxy for total phosphorus in seven of the 10 streams. The range of the annual mean total phosphorus concentration was compared with the environmental objective (good water quality) in each river. The results highlight that when the estimated mean total phosphorus concentration is close to the concentration of the environmental objective, an infrequent sampling strategy can lead to false water quality classification. .

**Supplementary Information:**

The online version contains supplementary material available at 10.1038/s41598-026-44177-1.

## Introduction

The European Commission’s Water Framework Directive (WFD)^1^ is the common water management legislation for member states of the EU as well as other European countries, like Norway. According to the directive, all water bodies are classified into one of five ecological status categories (High, Good, Moderate, Poor, or Bad) where the environmental objective is the Good/Moderate (G/M) boundary. The assessment of status is based on both biological indicators and water quality data. Water quality is given threshold levels based on the annual arithmetic mean concentrations of nutrients, such as total phosphorus (TP) or total nitrogen (TN), and these are used as a support to the biological indicators^2^. The Common Implementation Strategy guidelines^3^ advice that if the biological indicators suggest “Good status”, but the annual mean concentration of a nutrient is worse than good, then the overall ecological status of the water body should be degraded to Moderate. When a water body falls below the G/M boundary, the water managers should ensure that mitigation measures are implemented so the water body achieves at least Good ecological status. Mitigation measures can be expensive, and it is therefore essential to ensure that the nutrient concentration status is as correct as possible.

While this highlights the importance of an accurate estimate of annual mean concentration, it is a well-known challenge that the concentration of especially particle-associated substances in streams can vary dramatically over short periods of time^4,5^. Hence, the question arises as to how often water samples should be collected to obtain a reliable mean concentration. To date, some recommendations regarding the sampling frequency based on a single or a few streams have been made, but no general rule has been established. For example, a study was done in four English streams to assess the uncertainty of the mean annual concentrations of dissolved phosphorus, dissolved oxygen, pH, and water temperature for classification according to the WFD^6^. The authors found that in some cases, monthly sampling during a year could result in the same water body being assigned to three or four different WFD classes, whereas weekly sampling could reduce this variability to one or two different classes. In another study in small headwater streams, the number of samples required per month to obtain mean seasonal (April/May–September/October) concentrations within an error of 20% varied from 2 to 25 for turbidity, 2 to 39 for TP, and 1 to 16 for TN, corresponding to a maximum of 300, 468, and 192 annual samples, respectively^7^. Detecting trends in annual concentrations is also of importance to water managers, and an investigation of the statistical power of monthly samples of dissolved reactive phosphorus data from streams in New Zealand, showed that for long-term periods of 20 years, more than 95% of all monitored sites had sufficient samples to detect changes in nutrients. However, to detect changes within a five-year period would have required a fivefold increase in the sampling frequency^8^.

Although this demonstrates the value of sampling frequently, many monitoring programmes in Europe are still based on samples collected fortnightly, monthly, or even less often^9,10^. This is linked to economic considerations, since water sampling and laboratory analyses can be costly. It is therefore useful to determine how many samples per year are needed to estimate the annual mean concentration within a given level of uncertainty. A possibility to study the variation of concentrations in streams in more detail has been acquired through the development of sensor technology^4,10,11^. Sensor-based monitoring of turbidity offers frequent datasets at a relatively low cost. Turbidity is a water quality parameter related to the opaqueness (cloudiness) of water. Although turbidity is not part of the WFD classification, it is a much-used parameter and relatively easy to monitor, both in laboratories and by sensors. Hence, turbidity has been used as a proxy for suspended sediments and several sediment-associated elements, such as total phosphorus, metals and PAHs (^12–16^).

In this study, we first estimated the uncertainty in annual arithmetic mean turbidity when using three different sampling strategies often applied in national or regional monitoring programmes: weekly, fortnightly, and monthly. We used hourly sensor turbidity data from 10 Nordic streams and hypothesised that any between-stream variations in uncertainty might be explained by characteristics of the streams and their catchment areas. Furthermore, we hypothesised that the uncertainty in estimating annual mean turbidity could help quantify the uncertainty in estimating the annual mean concentration of TP.

## Materials and methods

The study is based on existing in-situ sensor stream turbidity data series and grab water samples derived from different monitoring programmes in four countries (Denmark, Finland, Norway, and Sweden). While the methods applied are largely consistent across sites, minor variations exist and are described below, the full details are provided in the supplementary material.

### Study sites and water quality data

Data from 10 streams in the four countries were selected based on the availability of in-situ turbidity sensor monitoring and water sampling data for TP that were used for calibration. Three years of data were used from each stream, ideally representing a dry, wet, and hydrologically average year. However, we also favoured years with minimal data gaps. The selected cases represent a variety of catchment sizes, land use distributions, predominant soil characteristics, turbidity levels, hydrological conditions, and WFD environmental objectives (Table 1).

An overview of the sensor brands used to measure turbidity is given in the supplementary material (Table S-1). The use of different sensor brands was inevitable, as we used data from on-going monitoring programmes. Different institutes tend to use different brands, depending on various criteria including the suitability of the sensor in the streams to be monitored. As noted by Skarbøvik et al.^14^ there is a need for harmonisation, but meanwhile the best option is to assess the impact of using different sensor brands for the uncertainty.

Sensor data were quality-controlled by the respective institutes according to their standard procedures (see^14^ for details). Hourly sensor measurements were used from all stream stations. Missing values were linearly interpolated if the number of consecutive gaps did not exceed 48, which corresponds to two days of measurements. Longer gaps were not interpolated but left as missing values. Interpolation was done using the R package ‘chillR function interpolate_gaps’ ^17^.

**Table 1:** Case streams’ catchment areas, long-term mean water flow (LTM), main land uses, WFD river types, classification boundaries, and main soil types.

Country	Stream name	Catchment area	LTM	Land use (%)			WFD river type	WFD environmental classification for TP (µg TP/l)					Main soil types^4^
		km^2^	l/s km^2^	Agric	Forest	Other	All are lowland rivers	Reference condition	High/ Good	Good/ Moderate	Median/ Poor	Poor/ Very Poor	
DK	Horndrup	5.48	9.2	70	19	11	RCB-5^1^			106^3^			Sandy loam (98.8%), Organic/Peat (1.2%)
DK	Lyby-Grønning	11.3	5.2	84	2	14	RCB-5^1^			106^3^			Sandy loam (47%), Clayey loam (52%)
FI	Aurajoki	756	9.4	37	51	12	Clay river, medium	< 40	40	60	100	130	Morain (54%), clay-rich (28%), organic/peat (11%)
FI	Hirvijoki	148	12.9	23	72	5	Clay river, medium	< 40	40	60	100	130	Clay (51%), Coarse sand with clay (25%), Gyttja/peat (10%), Coarse sand (6%), Fine sand (5%)
FI	Lepsämänjoki	22	10.4	37	53	10	Clay river, small	< 40	40	60	100	130	Clay (63%), Coarse sand with clay 9%, Gyttja/peat 8% Coarse sand 6%, Fine sand with clay 6%, Fine sand 6%
NO	Mørdre	7.7	8.4	65	28	7	Clay river, small			50			Silty clay (65%), ca. 35% morain in upstream parts
NO	Skuterud	4.5	14.4	61	29	10	Clay river, small			80			Silty clay (60%), ca. 40% morain in upstream parts
SE	Hågaån	121	7.6	31	55	14	R-07^2^	30	43	61	101	152	Morain (33%), clay (23%)
SE	Skivarp	122	6.4	77	9	9	R-07^2^	22	32	45	75	112	Clay (47%), sandy soils (27%)
SE	Sävjaån	746	7.6	34	57	9	R-07^2^	27	38	53	89	133	Morain (41%) clay (24%)

^1^ RCB-5: Lowland, Large, moderate-high alkalinity.

^2^ R-07: Lowland, < 10 000, organic and calcareous. (Lowland (< 200 m) high alkaline (> 1 mekv/l) and humic (> 30 mgPt/l).). May be transferred to a new typology class, Clay rivers, but this has not yet been done.

^3^ The mean of a span of 68–136 µg TP/l. The boundary is based on Lyche Solheim et al. (2024) https://projects.au.dk/fileadmin/projects/nordbalt-ecosafe/Filer/D1_2_FactSheetsWithRefValuesAndGMboundaryValuesDraft.pdf since DK has not yet set class boundaries for streams.

^4^ For larger streams, the soil type close to the monitoring station is given.

Water grab samples for laboratory analyses of TP were collected manually, using automatic samplers, or through a combination of both methods. There was some variation in the sampling strategies across the monitoring programmes (Supplementary material, Table S-2), but all datasets from the different streams included at least 65 TP samples for calibration purposes. In a comparison of 31 streams in Northern Europe, it was found that at least 70 samples for calibration yielded an R^2^ value above 0.6 between turbidity and suspended particulate matter^14^.

TP measurements were analysed by accredited laboratories according to nationally approved standards (Supplementary material, Table S3).

### Data analysis

To assess uncertainty of sampling strategies, mimic data series were generated for all streams by extracting subsets of the hourly sensor turbidity data using different sampling frequencies. A Monte Carlo approach was applied^18^, simulating different monitoring regimes where samples were randomly chosen within the boundaries of a week, 14 days, or a month. For each sampling strategy, 1,000 datasets of measurements were randomly constructed, and annual mean concentrations were calculated. These were then compared to the annual mean concentrations derived from the full hourly sensor data (named the “true” annual mean in this study). The percentage of uncertainty for each sampling strategy was calculated using the ChillR package. Total uncertainty (in %) was then calculated as shown in Eq. 1:

1 $$U\left( \% \right){\text{ }} = RMSE/TrueM*100$$

Where U is the total uncertainty (%) of finding the annual mean turbidity; RMSE is the root mean square error of prediction; and TrueM is the ‘true’ annual arithmetic mean turbidity value as found from hourly observations.

Next, we selected the following potential factors that might explain variations in total uncertainty between the streams (data provided in Table 1, and Supplementary material Tables S-4 and S-5):

- Size of catchment area (km^2^).
- Proportion of agricultural area in the catchment (%).
- Proportion of forested area in the catchment (%).
- Discharge per catchment area and year (l/s km^2^).
- Flashiness index (RBI).
- Indicators that expressed the range of turbidity: The maximum value, the 98- and 95-percentiles, and the standard deviation (NTU or FNU).
- Ratio of turbidity outliers to the number of turbidity records per year.

For the flashiness of the water flow, we used the Richards-Baker flashiness Index (RBI)^20^, which we calculated using the ContDataQC in R package^21^. The RBI was calculated per year, for each of the three years with data in each stream (Supplementary material Table S-4).

For the range in turbidity values, the lower values for all streams were zero or near zero, and we therefore tested for the maximum value, the 95 and 98 percentiles. The two latter were included because the maximum turbidity in some cases can equal the upper limit of the sensor’s detection range and is often reached in only a few spikes per year, possibly due to instrument error.

We fitted a linear model using the R program and assessed it for multicollinearity using variance inflation factors (VIF), and for normality of residual distribution using the Shapiro-Wilk test and residual plots. Some of the parameters were log10-transformed to improve the visual representation in the graphs (catchment area, discharge per unit area, and percentiles of turbidity).

The full dataset for the linear model consisted of 90 observations (10 streams, 3 years, 3 sampling strategies). For model development, half of the data was randomly selected (45 observations) and the remaining half was used for validation (45 observations). This process was repeated four times to ensure that the random selection did not include outliers or other influential data that could affect the results.

Finally, tests were done on the impact on uncertainty of using different sensors. Whereas all other parameters that we tested against had a natural range, the sensor brands were given a factor value from 1 to 5 (see Supplementary material Table S1 and Figure S3). For this test, we used a Kruskal-Wallis test^19^ as it is better suited for testing if samples originate from the same distribution.

For all streams, the TP concentrations analysed from grab samples were used to establish a linear regression equation between sensor-based turbidity concentrations from the same time the grab-samples were collected. We evaluated model performance using p-values and R^2^ values, and also closely examined the correlation plots (Supplementary material Figure S-2). Based on this assessment, we decided an R^2^ of 0.6 or higher to indicate an acceptable correlation to transform the turbidity concentrations into TP concentrations. For the streams with acceptable correlations, we calculated the annual mean TP-values based on weekly, fortnightly, and monthly sampling strategies and compared the result with the WFD environment objective for each stream.

## Results

### Uncertainty in annual mean turbidity

**Fig. 1:**
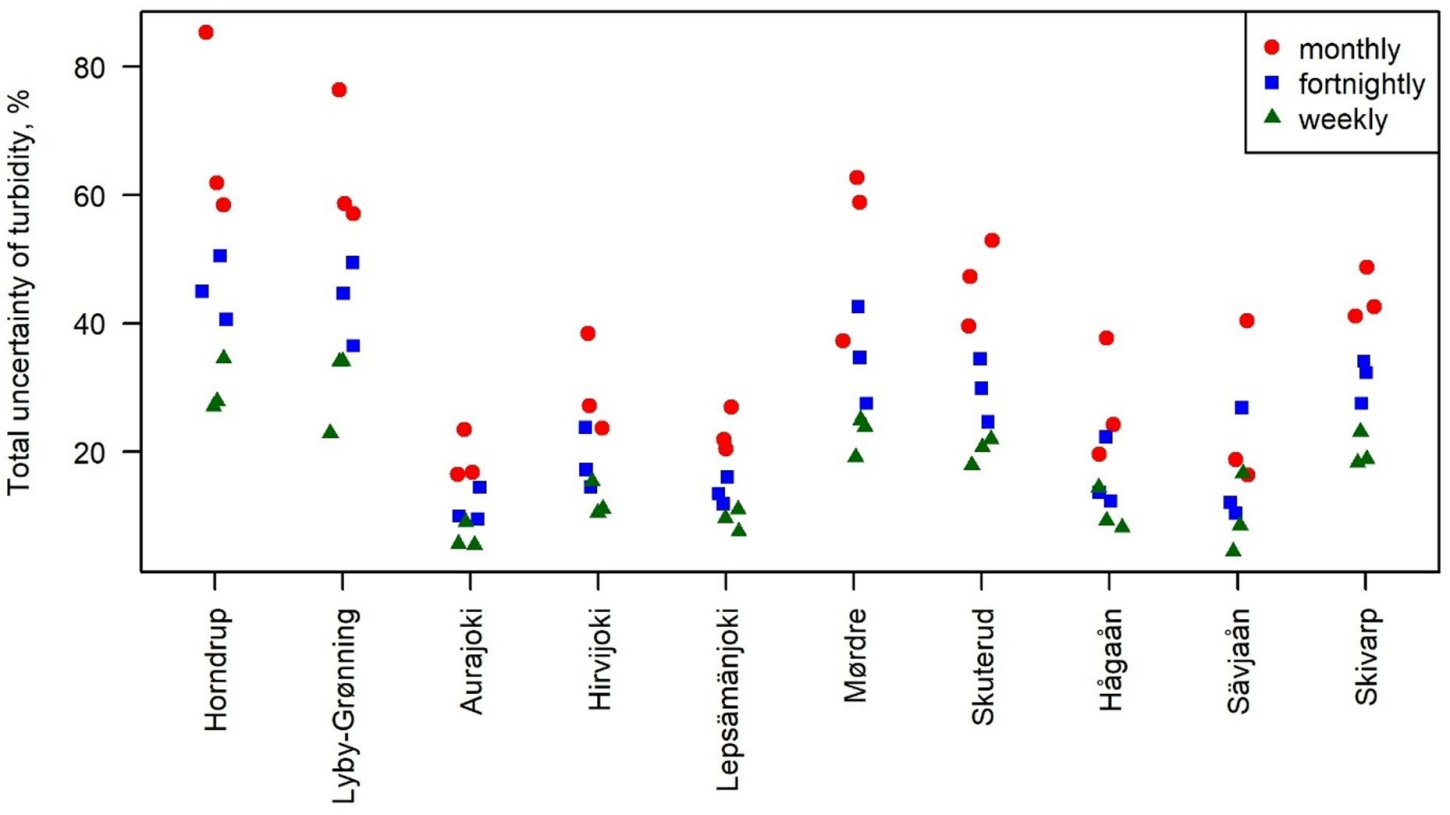
Uncertainty of calculating annual mean turbidity in the 10 rivers for three years each (the red, blue, and green dots represent monthly, fortnightly, and weekly sampling strategies, respectively).

In general, the uncertainty in the annual mean turbidity, as compared to the ‘true mean’, decreased with increasing sampling frequency (Fig. 1). On average for all streams, weekly sampling gave an uncertainty of 17 ± 9% (mean ± standard deviation with a range of 4.5%-35% (max. and min.)), fortnightly sampling of 26 ± 13% (10% – 51%) and monthly sampling of 40 ± 19% (16%–85%).

However, there were variations in the range of uncertainty between the 10 streams. The highest levels of uncertainty were found in the two Danish streams, and the lowest levels were found in the three Finnish streams and the Swedish Hågaån (Fig. 1).

We also found that, on average for 1,000 runs, there was a larger risk of underestimating the mean turbidity. The proportion of runs where estimated mean turbidity was lower than the ‘true mean’, was, on average, 58% for weekly sampling, 60% for fortnightly sampling, and 65% for monthly sampling. If turbidity can be expressed as a proxy for other substances such as heavy metals or TP, it follows that managers may assume that the state of a river water body is in a better state than it is.

### Factors affecting the variation in uncertainty

To investigate possible reasons for the variation in the uncertainty between the streams, the uncertainty was compared with the stream and catchment factors listed in the method’s chapter. For each of these factors, the R^2^ and p-values are shown in Supplementary material Table S6 and Figure S1. The best correlations were found for land use percentage of agricultural (R^2^ = 0.43) or forested (R^2^ = 0.39) land, catchment size (R^2^ = 0.35), and sampling frequency (R^2^ = 0.31). The proportion of agricultural vs. forested land was intercorrelated in our catchments, as they were predominantly rural.

From this, we developed a multiple linear model where uncertainty of finding annual mean turbidity could be described as follows:

2 $$U = 10^{ \wedge } \left[ {1.3812 - \left( {0.0095^{*} \cdot n} \right) - \left( {0.0003^{*} \cdot Cz} \right) + \left( {0.0066^{*} \cdot A} \right)} \right]$$

where U is the total uncertainty of finding ‘true mean’ turbidity; n is the number of samples for the given sampling strategy, Cz is catchment size and A is the % area of agricultural land. The equation was also tested with the other factors listed in the Method’s section (e.g., discharge, flashiness index, turbidity span, number of outliers), but the correlation did not improve by using any of these additional factors.

To determine the number of samples needed to stay within any given level of uncertainty (U), the same equation can also be expressed as:

3 $$n = {\text{ }}[1.3812 - \log 10\left( U \right) - \left( {0.0003*{\text{ }}Cz} \right) + \left( {0.0066*{\text{ }}A} \right)]/0.0095$$

**Fig. 2:**
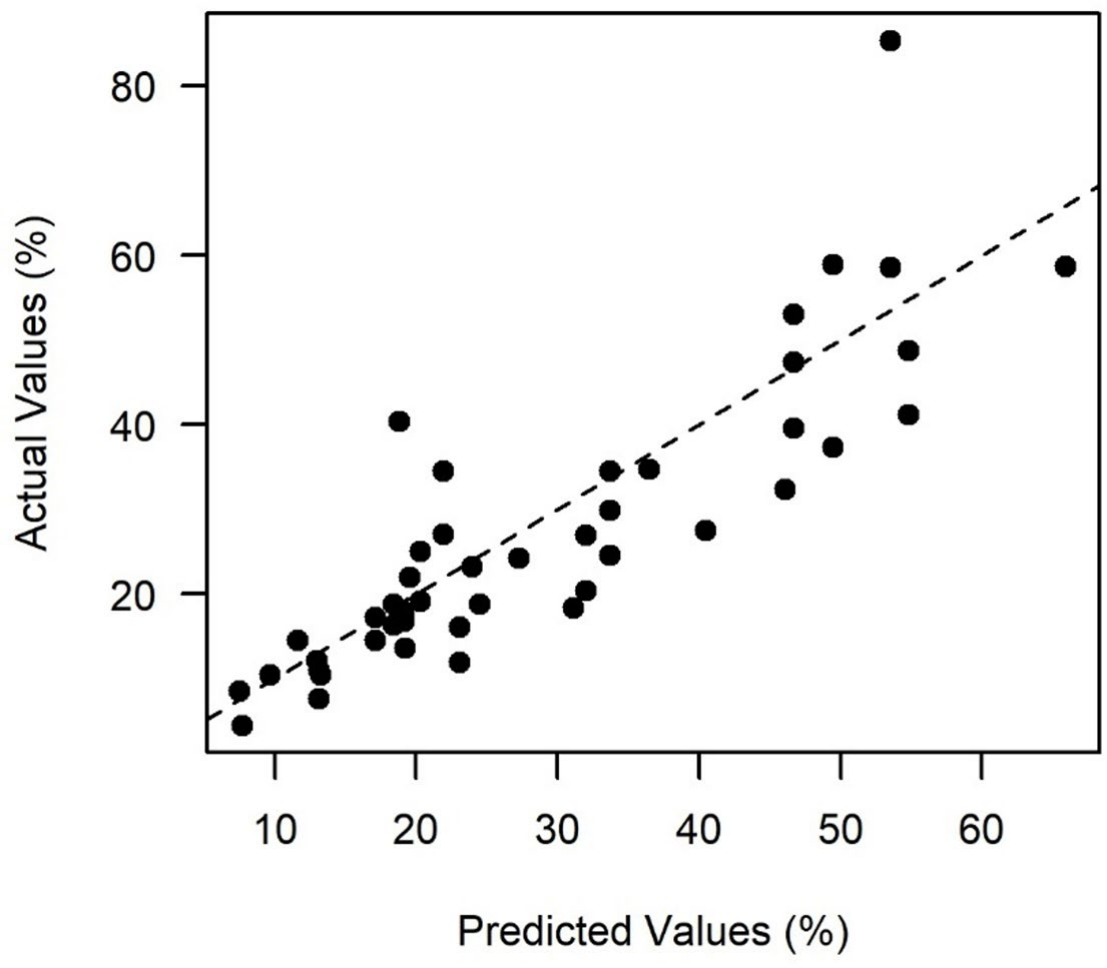
Predicted vs. actual values (%) in the uncertainty model (Eq. 2) for finding annual mean turbidity, including catchment size, proportion of agricultural land, and number of samples per year, for the 45 observations not used in the modelling. Dashed line is the 1:1 line; R^2^: 0.79.

The VIF scores of the parameters used were below 5 for all predictions, indicating no multicollinearity concern. The model was significant, *p* < 0.001 and explained 79% of the variance in the total uncertainty (adjusted R^2^ = 79) (Fig. 2). Supplementary material Table S-7 provides further details on the model.

**Fig. 3:**
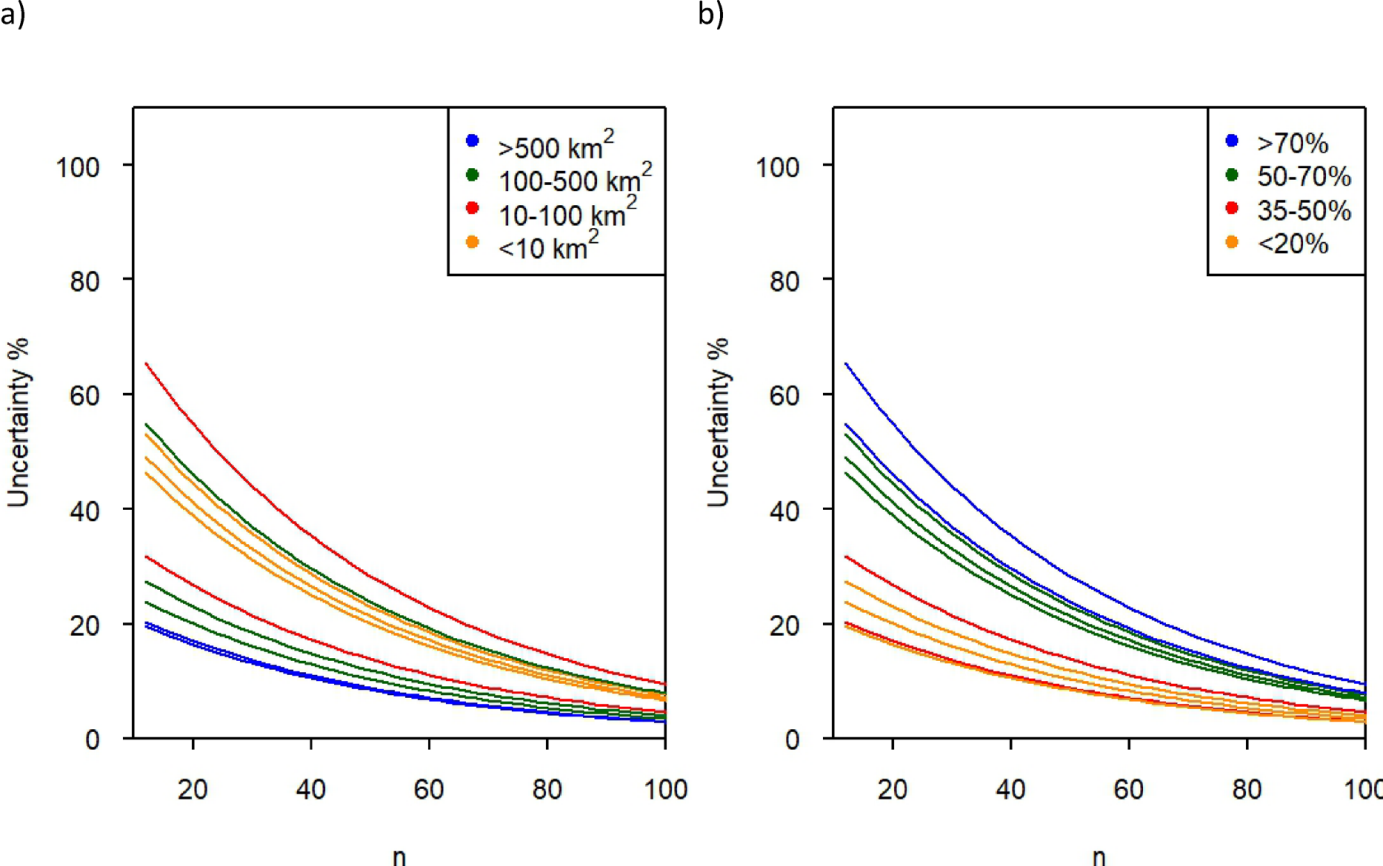
Uncertainty (%) (y-axis) of finding the annual ‘true mean’ turbidity as a function of the number of samples per year (n; x-axis), depending on catchment size (**a**) and the proportion of agricultural land in the catchment (**b**). Each graph represents one of the 10 studied streams. (**a**) Catchment size. (km2) (**b**) Land use, (% agricultural land in catchment)

We then fed the data from our 10 streams into Eq. 3, and the result is shown in Fig. 3a (number of samples for any given uncertainty depending on catchment size) and Fig. 3b (similar but depending on the proportion of agricultural land in the catchments). The figures illustrate that larger rivers may be sampled less often than smaller streams within the same uncertainty level, but at the same time, a large agricultural river may need the same number of samples as a smaller, forested stream. As an example, according to this model, in a river of 800 km^2^ catchment area and only 20% agricultural land, 29 samples a year would be sufficient to give a mean annual turbidity within 10% uncertainty, whereas a stream of 10 km^2^ catchment area and with 80% agricultural land would need 95 samples to achieve the same level of uncertainty.

The impact on the uncertainty of different sensor brands was also tested, but it should be noted that this test mainly reflects the differences between the four countries, as each country used one sensor brand each (or two very similar ones for the Finnish cases); see Table S1 in the Supplementary materials. Each sensor type was given a number from 1 to 5, which means that the test was done on factors and not continuous data series. The streams that were most similar in terms of land use and catchment size, were the Swedish and two of the Finnish on the one hand (relatively large); and on the other hand, the Norwegian and Danish streams (smaller and all of them with more agriculture than forests), cf. Table 1. The last stream, Finnish Lepsämänjoki, was a smaller stream, with more forest than agriculture. It was assumed that if the uncertainty differed significantly based on which sensor brand was used, then we would see significant differences between the larger streams in Sweden and Finland on the one hand, and the Norwegian and Danish streams on the other hand. The test revealed no significant difference between the uncertainty in the five larger streams in Sweden and Finland, or the four smaller streams with high percentage of agriculture in Norway and Denmark (Figure S3, Supplementary material). In other words, for streams of relatively similar land use or catchment size, the use of different sensors did not give significant differences in uncertainty. Certainly, the impact of the use of different brands should be further explored in other studies, preferably with different sensors in the same stream, but more investigations to this end were beyond the scope of this study.

### Uncertainty in TP concentrations and implications for WFD implementation

Of the 10 streams in this study, seven had correlations between turbidity and TP that were deemed acceptable (Table 3). The three streams with poorest correlations, Mørdre, Hågaån and Skivarp, were omitted from the following analyses.

**Table 3:** Correlations (R^2^ and p-values) between turbidity and TP for the 10 streams. The three streams with names in italic were not used in the assessment of the impact on sampling frequency on annual mean TP.

Country	Name of stream	TP vs. turbidity		Number of water samples
		*R* ^2^	*p*-value	
DK	Horndrup	0.92	7.9 E-63	86
DK	Lyby-Grønning	0.81	4.7 E-32	107
FI	Aurajoki	0.77	1.1 E-35	65
FI	Hirvijoki	0.82	4.4 E-25	211
FI	Lepsämänjoki	0.85	5.6 E-87	155
NO	*Mørdre*	0.53	7.5 E-27	214
NO	Skuterud	0.66	3.2 E-51	137
SE	*Hågaån*	0.40	2.0 E-16	125
SE	Sävjaån	0.65	2.4 E-24	113
SE	*Skivarp*	0.25	3.7 E-09	100

**Fig. 4:**
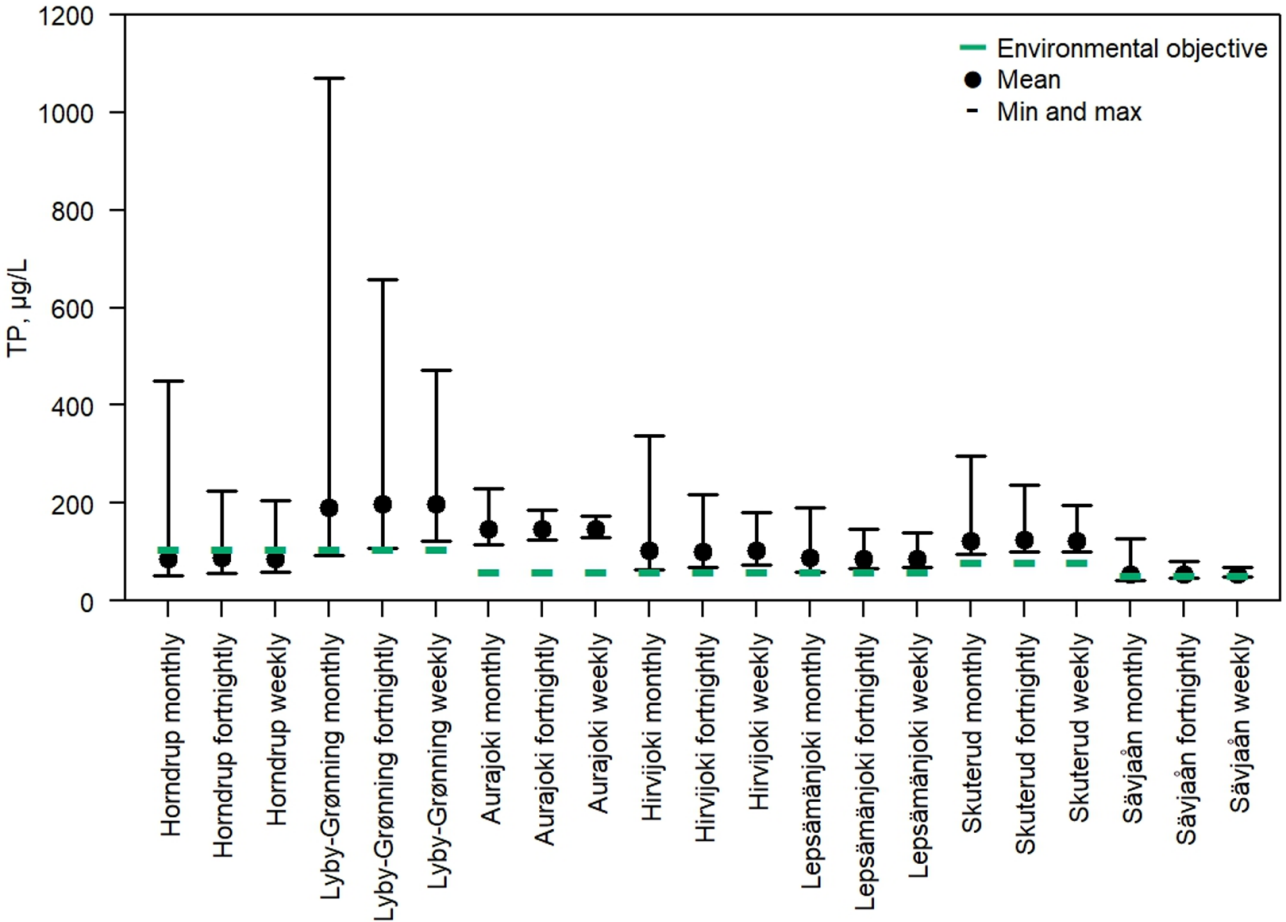
Environmental objectives (green horizontal lines, denoting the TP concentration between moderate and good water quality status) compared to the range between maximum and minimum estimated average TP-concentrations (black horizontal lines), with the average TP-concentration of 1000 runs shown as black dots. Three different sampling strategies are demonstrated for each river.

For the seven streams with acceptable correlations, we calculated the annual mean TP-values from the correlation equations between turbidity by sensor and TP by water sample analyses, for weekly, fortnightly, and monthly sampling strategies. Figure 4 shows the span in annual mean concentration of TP together with the mean concentration of TP for all 1000 runs. The latter value largely coincided with the ‘true mean’ found by hourly data (derived from the correlation between TP and turbidity), and the ‘true mean’ is therefore not shown in the figure. The environmental objectives of TP (green horizontal lines) are not the same for all streams, since different stream types have been assigned different good status objectives, determined during geographical intercalibration exercises throughout the European states that have ratified the WFD^2,22,23^.

For monthly sampling, the range between the estimated lowest and highest annual mean TP concentration varied from 86 µg/l in the Swedish Sävjaån to as much as 979 µg/l in the Danish Lyby-Grønning, and the average range for the five remaining streams amounted to 223 µg/l. This means that a rather huge risk is involved in using monthly sampling to find the annual mean TP-concentration. For fortnightly sampling, the TP-concentrations in the seven streams had a range from 34 µg/l in Sävjaån, to 550 µg/l in Lyby, with an average for the remaining five streams of 120 µg/l; and for weekly sampling the range varied from 21 µg/l in Sävjaån to 350 µg/l in Lyby, with an average of 92 µg/l for the other five streams. As shown in the graph of Fig. 4 and in Table 1, the environmental objectives (G/M boundaries) of these streams vary from 45 µg/l in Sävjaån to 106 µg/l in Lyby-Grønning and Horndrup. The range in annual mean concentration estimates was in general higher than the environmental objective for monthly and fortnightly sampling, and even for weekly sampling, the range in annual mean concentration exceeded the environmental objective in five of seven streams.

## Discussion

Our initial hypothesis was that hourly sensor turbidity data could be used to assess the uncertainty of different sampling frequencies in estimating the annual mean turbidity and, through correlations, the annual mean concentration of TP. We also hypothesized that the uncertainty may be impacted by stream and catchment characteristics. We found that the uncertainty increased with decreasing sampling frequency, and that the variation in the uncertainty between streams was best explained by the catchment size and land use. At the same time, factors that express different discharge and turbidity characteristics had very low impact on the uncertainty. This knowledge is important for designing effective sampling strategies to assess whether streams meet the requirements of the WFD.

The effect of catchment size may be explained by differences in temporal variation in water flow, and therefore also turbidity and particle-associated substances in larger vs. smaller streams. Small streams often respond rapidly to rain or snow-melt events, with ensuing increases in water flow and turbidity. Similarly, to estimate suspended particulate matter fluxes within 20% uncertainty, it was found that the river Garonne would need to be sampled every third day, whereas a smaller river would have to be sampled as often as every seventh hour^24^.

Our model also showed that the uncertainty of the mean annual concentration is higher in agricultural catchments than in catchments dominated by forest cover. In Nordic catchments it has been documented that the range in TP concentrations is much higher in agricultural than in forested catchments^25^. The soil types in Nordic catchments dominated by agriculture are likely to be more fine-grained than in catchments dominated by forest, where there often are coarser moraine deposits^14^. Moreover, in agricultural catchments the seasonal changes in vegetation cover will leave the soil more vulnerable to erosion. There may also be fewer trees along the riverbanks and therefore more bank erosion in agricultural areas^26^. All of this can result in higher concentrations and thereby greater variations in the concentrations of turbidity and TP, making it more difficult to estimate a reliable annual mean concentration.

The risk of getting a wrong classification increases as the water body approaches the borders between the status classes^6^; and when the water body is close to the environmental objective an erroneous classification can give rather large economic implications. If the water body is still in moderate state, mitigation measures to improve it need to be implemented, whereas if the water body has reached the environmental objective, the measures merely need to ensure that the status does not deteriorate. In cases such as the Aurajoki and Skuterud streams, where the annual mean concentration was significantly higher than the environmental objective, regardless of sampling frequency, it may not be cost-effective to increase the monitoring budgets to improve the estimate of the annual mean, but better to investigate which measures should be implemented to improve the water quality. On the other hand, a less accurate monitoring strategy would make it more difficult to calculate the need for mitigation measures, and to track whether implemented measures work as planned. As stated by the EC ^27^: *“The cost of measures for improvement in water status would be orders of magnitude greater than the costs of monitoring. The extra costs of monitoring to reduce the risk of misclassification might therefore be justified in terms of ensuring that decisions to spend larger sums of money required for improvements are based on reliable information on status.”* In practice, however, there will likely be a trade-off between the costs for monitoring vs. the costs for measures, not least since the funding may come from different budget sources and sectors.

The sources of errors in the data used in this study relate mainly to the reliability of the different sensors used and to the relationship between turbidity and the TP concentration. Kahiluoto et al. ^28^ found that turbidity sensor measurement had an uncertainty of 11–27% (k = 2) for the range of 5–40 FNU. As far as we know, there is no set rule for how good a correlation should be in order to use turbidity as a proxy for another substance. Lannergård et al. ^12^ presented an overview of the R^2^s between turbidity and TP from eight different studies, varying from 0.25 to 0.90, but with most correlations showing an R^2^ above 0.6. Minaudo et al. ^29^ showed that a nonlinear model between turbidity and TP can sometimes be more feasible than a linear regression model. Moreover, in three Finnish streams the linear correlations between turbidity from sensors and TP from grab samples had R^2^ values varying between 0.77 and 0.80, but if a few high TP concentrations were omitted, the R^2^ was reduced to 0.57–0.74 ^13^. This illustrates that R^2^ alone is insufficient evidence for correlations, and that consideration should be given to correlation graphs to check for single high values, as well as to other statistics. In our case, this was achieved through the graphs shown in the supplementary material, Figure S-2, and when analysing the impacts of sampling frequency on TP annual means, we only used the streams with an R^2^ between turbidity and TP above 0.65. It was beyond the scope of the work to assess the reason for poorer correlations in three of the streams, but it can be assumed that such variation can be linked to other sources of turbid water, like high amounts of organic matter, or high levels of dissolved P, from sources such as sewage or animal manure.

In the years to come, the economic costs of implementing the WFD may become an increasing challenge in a world facing uncertain economic developments due to ongoing military conflicts and potential trade wars, and a possible subsequent need to intensify food production locally^30^. At the same time, eutrophication is a challenge that can be expected to increase in magnitude in the future due to climate change^31,32^. Hence, it will be of great importance to choose the most cost-effective monitoring strategy to ensure that streams under the WFD are properly managed.

## Conclusions

By using hourly turbidity measurements from 10 Nordic streams, we found that the uncertainty in estimating annual arithmetic mean turbidity increased with decreasing sampling frequency, decreasing catchment size, and increasing proportion of agricultural land. Based on these findings, we developed an equation that can be used by managers and scientists alike, to direct their monitoring programmes towards the optimal sampling frequency for streams with varying catchment size and land use, given a chosen level of uncertainty. This equation can be refined further as more data from additional streams become available. In our case, seven of the 10 streams had a good correlation between turbidity and TP, and for these streams also uncertainty in TP could be calculated. This highlighted that when the estimated mean total phosphorus concentration is close to the concentration of the environmental objective, an infrequent sampling strategy may lead to false water quality classification.

Moreover, we observed a higher risk of underestimating than overestimating mean annual turbidity. If we transfer this finding to other particle-associated substances for which turbidity can potentially act as a proxy, such as TP, it suggests that managers may believe their water bodies are in a better state than they actually are.

Deciding whether to use resources to improve the accuracy of annual mean concentrations by increasing the monitoring frequency, or to implement mitigation measures, may depend on the state of the water body. In streams where the environmental state is significantly poorer than the environmental objective, regardless of sampling strategy, resources might be better spent on reducing nutrient losses. The drawback with this strategy is that it can be challenging to determine the level of measures needed and whether the implemented measures have contributed to an improved water quality.

## Supplementary Information

Below is the link to the electronic supplementary material.

Supplementary Material 1

## Data Availability

Data is provided within the manuscript and in the supplementary material. Raw data from turbidity sensors may be accessed by contacting the authors.
