# Supplementary material for: High-frequency turbidity by sensors as a proxy for total phosphorus: implications of sampling strategies on the water framework directive classification
Source: Sci Rep. 2026 Mar 12;16:13317. doi: 10.1038/s41598-026-44177-1 (PMC13106753; doi:10.1038/s41598-026-44177-1)
Supplement: Supplementary file 1 — Supplementary Material 1 [file 41598_2026_44177_MOESM1_ESM.docx]

**Supplementary materials**

**High-frequency turbidity by sensors as a proxy for total phosphorus: Implications of sampling strategies on the Water Framework Directive Classification**

Eva Skarbøvik^(a)^, Anastasija Isidorova^(a)^, Maria Kämäri^(b)^, Pasi Valkama^(b)^, Sofie G.M. van’t Veen^(c)^, Emma E. Lannergård^(d)^, Jens Fölster^(d)^, and Brian Kronvang^(e)^

^a^Norwegian Institute of Bioeconomy Research (NIBIO), Division of Environment and Natural Resources, P.O. Box 115, 1431 Ås, Norway

^b^Finnish Environment Institute (Syke), Marine and freshwater solutions, Latokartanonkaari, 11, FI-00790 Helsinki, Finland

^c^Aarhus University, Department of Ecoscience, C.F. Møllers Allé, DK-8000 Aarhus C, Denmark and Envidan A/S, Vejlsøvej, 23, DK-8600 Silkeborg, Denmark

^d^Swedish University of Agricultural Sciences, Department of Aquatic Sciences and Assessment, PO Box 7050, 750 07 Uppsala, Sweden

^e^Aarhus University, Department of Ecoscience, C.F. Møllers Allé 3, DK-8000 Aarhus C, Denmark

**Corresponding author:** Eva Skarbøvik, Norwegian Institute of Bioeconomy Research (NIBIO), Division of Environment and Natural Resources, P.O. Box 115, 1431 As, Norway

Mail address corresponding author: [eva.skarbovik@nibio.no](mailto:eva.skarbovik@nibio.no)

Phone number: +47 416 286 22

**Supplementary tables**

**Table S1.** Sensors used in the study, with measuring technique and range used

|  | Sensor brand and type | Measurement technique | Range and accuracy  (NTU/FNU) | Test A | Test B |
| --- | --- | --- | --- | --- | --- |
| SE | YSI 600OMV-VS, YSI EXO2,  In Situ Aquatroll600 | Infrared light, scatter 90° | 0-1000;  0-4000 | 1 | 1 |
| NO | SEBA, MPS-D8 | Infrared light 90° | 0-3200 | 2 | 1 |
| FI  Aurajoki River  Hirvijoki River | S::CAN * Spectrolyser, optical path length 2mm | UV-VIS spectrometry, light absorbance (190-720 nm) | 0-4000  0-2500 ± 12 | 3 | 2 |
| FI  Lepsämänjoki River | S::CAN * Spectrolyser, optical path length 5 mm | UV-VIS spectrometry, light absorbance  (190-720 nm) | 0-4000  0-1250 ± 3 | 4 | 2 |
| DK | Hach, Solitax | Infrared light, 90° | 0.001-4000 | 5 | 1 |

* Measuring details: Xenon flash lamp, 256 photo diodes, automatic cleaning with compressed air before measurements, factory pre-calibrated.

**Table S2.** Methods for collecting water samples for calibration. NA: Not applicable

| Country | Institute/data series | Automatic sampler (ISCO) | Hand grab samples | Event sampling (high water discharges) | Sampling at regular intervals (indicate interval) | Sampling site: High turbulence of water | Sampling site: Low turbulence of water | Good routines/site to avoid that ISCO draws bed/saltation sediments | Depth of sampling below surface | Sensor is placed in a monitoring shed, water is pumped in |
| --- | --- | --- | --- | --- | --- | --- | --- | --- | --- | --- |
| DK | Lyby-Grønning | X | X | X | Monthly |  | X | yes | Ca. 15 cm | no |
| DK | Horndrup | X | X | X | Monthly |  | X | yes | Ca. 15 cm | no |
| FI | Aurajoki |  | X | X | No regular interval^1^ |  | X | NA | 40 cm | no |
| FI | Hirvijoki |  | X | X | Monthly + event |  | X | NA | 40 cm | no |
| FI | Lepsä-mänjoki |  | X | X | Event based |  | X | NA | 15-50 cm from sensor depth | no |
| NO | Mørdre | X |  | X | Event samples | (x)* |  | yes | Ca 40 cm | no |
| NO | Skuterud | X |  | X | Event samples | (x)* |  | yes | Ca 30 cm | no |
| SE | Hågaån |  | X | X | 14 days + events | X |  | NA |  | No |
| SE | Skivarps-ån |  | X | X | 14 days + events | X |  | NA |  | No |
| SE | Sävjaån |  | X |  | Monthly |  | X | NA |  | No |

* Sampled near V-weir at the most turbulent site.

**Table S3.** Analytical methods for determining TP.

| **Rivers** | **Reference for analytical method TP (and uncertainty if available)** |
| --- | --- |
| Horndrup  Lyby-Grønning | Danish standard DS/EN ISO 6878:2004 |
| Aurajoki,  Hirvijoki  Lepsämänjoki | SFS 3026 mod. DA (uncertainty 15 %) |
| Mørdre  Skuterud | Norwegian standard NS-EN ISO 15681-2:2018 (Uncertainty 20-40 %). |
| Hågaån  Skivarpsån  Sävjaån | SS-EN ISO 6878:2005 |

**Table S4. Average area-specific water discharge (l/s and km^2^) for the selected three years of monitoring in each stream, and long term means (LTM), including years used to calculate LTM.**

| C | Name | 2016 | 2017 | 2018 | 2019 | 2020 | 2021 | 2022 | 2023 | LTM | Years for LTM |
| --- | --- | --- | --- | --- | --- | --- | --- | --- | --- | --- | --- |
|  |  | - l/s/km^2^ - | | | | | | | | | From-to |
| DK | Horndrup |  |  |  |  | 10.0 | 8.6 | 8.2 |  | 9.2 | 1991-2020 |
| DK | Lyby |  |  |  |  | 7.0 | 3.6 | 3.9 |  | 5.2 | 1991-2020 |
| Fi | Aurajoki |  |  | 4.9 | 12.0 |  | 9.2 |  |  | 9.4 | 1994-24 |
| FI | Hirvijoki |  |  | 6.0 | 15.0 |  | 13.1 |  |  | 12.9 | 2017-22 |
| FI | Lepsämänjoki |  |  | 7.4 | 11.5 | 15.6 |  |  |  | 10.4 | 2006-2024 |
| NO | Mørdre |  |  |  | 12.6 |  | 8.2 | 3.5 |  | 8.4 | 1991-2020 |
| NO | Skuterud |  |  |  |  | 24.7 | 12.0 | 10.9 |  | 14.4 | 1991-2020 |
| SE | Hagaån |  |  |  | 9.2 | 4.5 |  |  | 14.5 | 7.6 | 1991-2020 |
| SE | Skivarp |  |  |  | 7.5 | 5.5 |  | 7.4 |  | 6.4 | 1991-2020 |
| SE | Sävjaån | 2.6 | 3.9 |  | 6.1 |  |  |  |  | 7.6 | 1991-2020 |

**Table S5.** Mean, maximum and standard deviation (StDev) of water discharge (Q), flashiness index (RBI); mean, maximum, standard deviation (StDev), outliers, 95 and 98 percentiles of turbidity (turb); and mean and maximum TP concentration in the water samples used for calibration. Data shown per year.

|  |  | **Water discharge factors** | | | | **Turbidity factors** | | | | | | **TP in water samples** | |
| --- | --- | --- | --- | --- | --- | --- | --- | --- | --- | --- | --- | --- | --- |
| **Name** | **Year** | **Mean**  **Q**  **l/s** | **Max**  **Q** | **StDev**  **Q** | **RBI** | **Mean**  **turb** | **Max**  **turb** | **StDev**  **turb** | **Outliers**  **turb** | **q95**  **turb** | **q98**  **turb** | **Mean**  **TP**  **µg/l** | **Max**  **TP**  **µg/l** |
| Horndrup | 2020 | 55 | 1732 | 95 | 0.29 | 18 | 1343 | 57 | 1209 | 57 | 127 | 93 | 4196 |
| Horndrup | 2021 | 48 | 835 | 51 | 0.28 | 17 | 821 | 40 | 1220 | 67 | 138 | 90 | 2581 |
| Horndrup | 2022 | 46 | 1187 | 66 | 0.24 | 12 | 511 | 26 | 817 | 36 | 77 | 75 | 1620 |
| Lyby-Grønning | 2020 | 80 | 1406 | 124 | 0.26 | 20 | 824 | 39 | 1046 | 77 | 128 | 164 | 3923 |
| Lyby-Grønning | 2021 | 42 | 446 | 50 | 0.26 | 38 | 1109 | 112 | 1168 | 143 | 383 | 250 | 5260 |
| Lyby-Grønning | 2022 | 45 | 700 | 77 | 0.26 | 20 | 963 | 46 | 1272 | 82 | 151 | 167 | 4577 |
| Hirvijoki | 2018 | 890 | 17310 | 1471 | 0,25 | 13 | 170 | 16 | 614 | 43 | 63 | 84 | 564 |
| Hirvijoki | 2019 | 2220 | 38200 | 4426 | 0.38 | 16 | 498 | 22 | 684 | 44 | 71 | 94 | 1564 |
| Hirvijoki | 2021 | 1932 | 26240 | 3045 | 0.32 | 28 | 828 | 26 | 686 | 68 | 87 | 130 | 2567 |
| Lepsämänjoki | 2018 | 162 | 2551 | 264 | 0.26 | 35 | 489 | 35 | 2045 | 101 | 150 | 81 | 630 |
| Lepsämänjoki | 2019 | 253 | 3643 | 374 | 0.29 | 34 | 1033 | 31 | 1061 | 84 | 111 | 79 | 1290 |
| Lepsämänjoki | 2020 | 343 | 3223 | 393 | 0.35 | 53 | 1022 | 52 | 1411 | 134 | 208 | 102 | 1277 |
| Aurajoki | 2018 | 3719 | 57521 | 6969 | 0.31 | 22 | 216 | 19 | 616 | 63 | 76 | 135 | 560 |
| Aurajoki | 2019 | 9062 | 112031 | 14325 | 0.32 | 28 | 436 | 32 | 637 | 91 | 120 | 149 | 1041 |
| Aurajoki | 2021 | 6971 | 64186 | 8641 | 0.32 | 34 | 215 | 23 | 338 | 75 | 91 | 163 | 557 |
| Mørdre | 2019 | 97 | 2257 | 209 | 0.63 | 167 | 3210 | 383 | 1101 | 594 | 1428 | 381 | 1881 |
| Mørdre | 2021 | 62 | 1466 | 116 | 0.48 | 138 | 3210 | 291 | 1083 | 521 | 1246 | 367 | 1881 |
| Mørdre | 2022 | 27 | 2212 | 100 | 0.74 | 110 | 2905 | 179 | 709 | 360 | 630 | 353 | 1731 |
| Skuterud | 2020 | 111 | 3339 | 238 | 0.68 | 62 | 2292 | 113 | 900 | 210 | 379 | 140 | 2038 |
| Skuterud | 2021 | 54 | 3059 | 162 | 0.52 | 41 | 1610 | 67 | 1258 | 156 | 199 | 122 | 1457 |
| Skuterud | 2022 | 49 | 3146 | 170 | 0.74 | 28 | 744 | 49 | 873 | 96 | 160 | 111 | 721 |
| Hågaån | 2019 | 1110 | 8199 | 1749 | 0.11 | 62 | 1291 | 84 | 479 | 183 | 305 | 89 | 847 |
| Hågaån | 2020 | 547 | 3285 | 559 | 0.10 | 35 | 460 | 30 | 613 | 99 | 129 | 73 | 334 |
| Hågaån | 2023 | 1759 | 14541 | 2510 | 0.13 | 39 | 386 | 40 | 749 | 109 | 179 | 75 | 289 |
| Skivarp | 2019 | 949 | 5824 | 1206 | 0.18 | 12 | 162 | 17 | 787 | 48 | 71 | 124 | 422 |
| Skivarp | 2020 | 671 | 5815 | 964 | 0.15 | 14 | 300 | 22 | 568 | 43 | 83 | 129 | 694 |
| Skivarp | 2022 | 900 | 7807 | 1452 | 0.16 | 17 | 620 | 36 | 849 | 62 | 124 | 134 | 1327 |
| Sävjaån | 2016 | 1928 | 7324 | 1922 | 0.05 | 17 | 449 | 27 | 356 | 37 | 62 | 58 | 609 |
| Sävjaån | 2017 | 2935 | 13550 | 3350 | 0.04 | 14 | 126 | 13 | 598 | 41 | 49 | 54 | 197 |
| Sävjaån | 2019 | 4585 | 31160 | 6488 | 0.06 | 11 | 76 | 15 | 997 | 50 | 62 | 50 | 133 |

**Table S6. Relationship between the total uncertainty of finding mean annual turbidity, and possible explanatory factors**

| **Possible explanatory factors (x in the equations in the next column). All were log10 transformed** | **Equation (y is the total uncertainty; x is the tested explanatory factor)** | **R^2^** | **p** | **Stats. Info^1^** |
| --- | --- | --- | --- | --- |
| Number of samples per year (n) | y=10^(x*-0.00935+1.64153) | 0.31 | 1.42E-08 | NND |
| Size of catchment area (km^2^) | y=10^(x*-0.20221+1.70088) | 0.35 | 9.76E-10 | ND |
| Proportion of agricultural area (%) | y=10^(x*0.00881+0.90449) | 0.43 | 1.63E-12 | ND |
| Proportion of forested area (%) | y=10^(x*-0.00788+1.65694) | 0.39 | 3.41E-11 | ND |
| Discharge per catchment area (l/s and km^2^) | y=10^(x*0.01506+1.3574) | 0 | 0.5 | NS |
| Flashiness index (RBI) | y=10^(x*0.38044+1.24591) | 0.07 | 1.25E-02 | ND |
| Turbidity 98-percentile (NTU/FNU) | y=10^(x*0.26894+0.77781) | 0.12 | 6.65E-04 | ND |
| Turbidity maximum level (NTU/FNU) | y=10^(x*0.33404+0.42717) | 0.25 | 6.98E-07 | ND |
| Turbidity 95-percentile (NTU/FNU) | y=10^(x*0.16224+1.04445) | 0.03 | 0.08 | NS |
| Ratio of turbidity outliers/number of records | y=10^(x*0.45126+0.04556) | 0.08 | 7.45E-03 | ND |
| The standard deviation of turbidity (NTU/FNU) | y=10^(x*0.34252+0.8062) | 0.20 | 1.07E-05 | ND |

^1^Statistical information: NND: Not normally distributed; ND: Normally distributed; NS: Not significant

**Table S7. Regression parameters, standard errors, t-values and p-values for Equation 2**

| Independent variable | Slope | Std. error | t-value | Probability |
| --- | --- | --- | --- | --- |
| n | -0.00945164 | 0.00124997 | -7.562 | <0.001 |
| Agricultural area % | 0.00660639 | 0.00101061 | 6.537 | <0.001 |
| Catchment area | -0.00030467 | 0.00007724 | -3.944 | <0.001 |
| Intercept | 1.38116544 | 0.07322614 | 18.862 | <0.001 |
|  |  |  |  |  |
| Multiple R2 | 0.8013 |  |  |  |
| Adjusted R2 | 0.7868 |  |  |  |
| F-statistic | 55.11 |  |  |  |
| SEE | 0.1337 |  |  |  |
| Observations | 45 |  |  |  |
| Probability (p-value) | <0.001 |  |  |  |

**Supplementary figures**

| 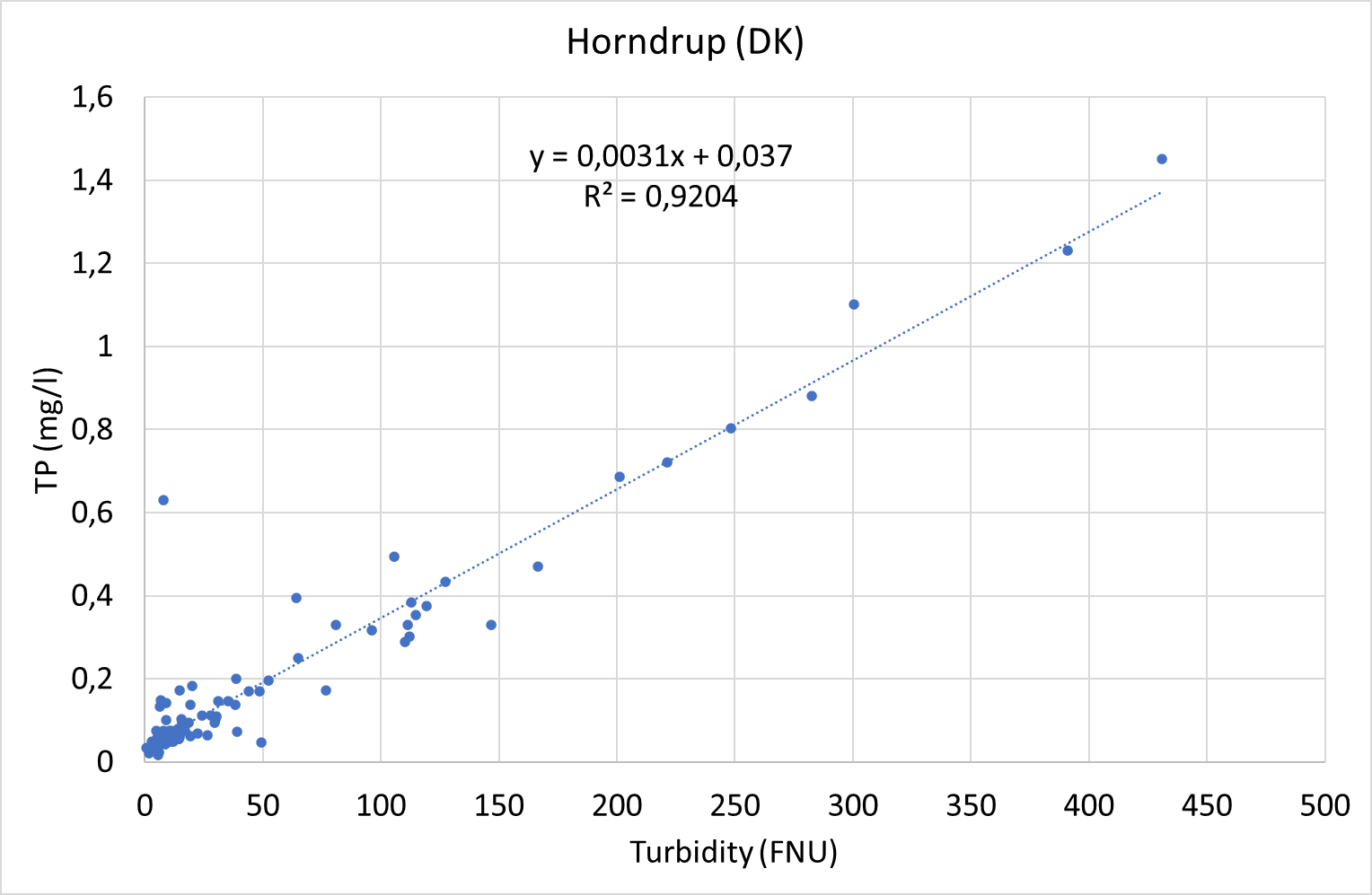 | 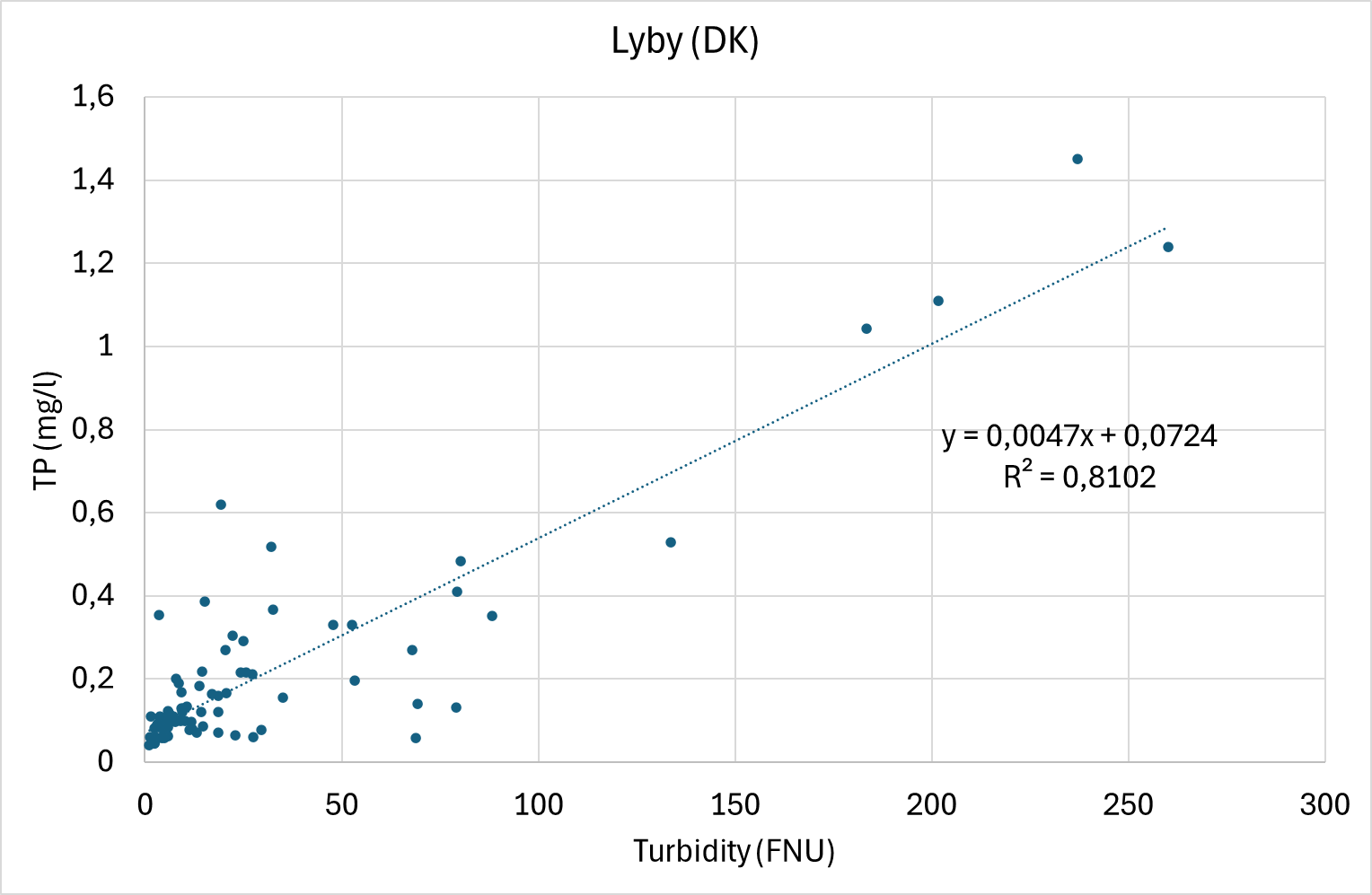 |
| --- | --- |
| 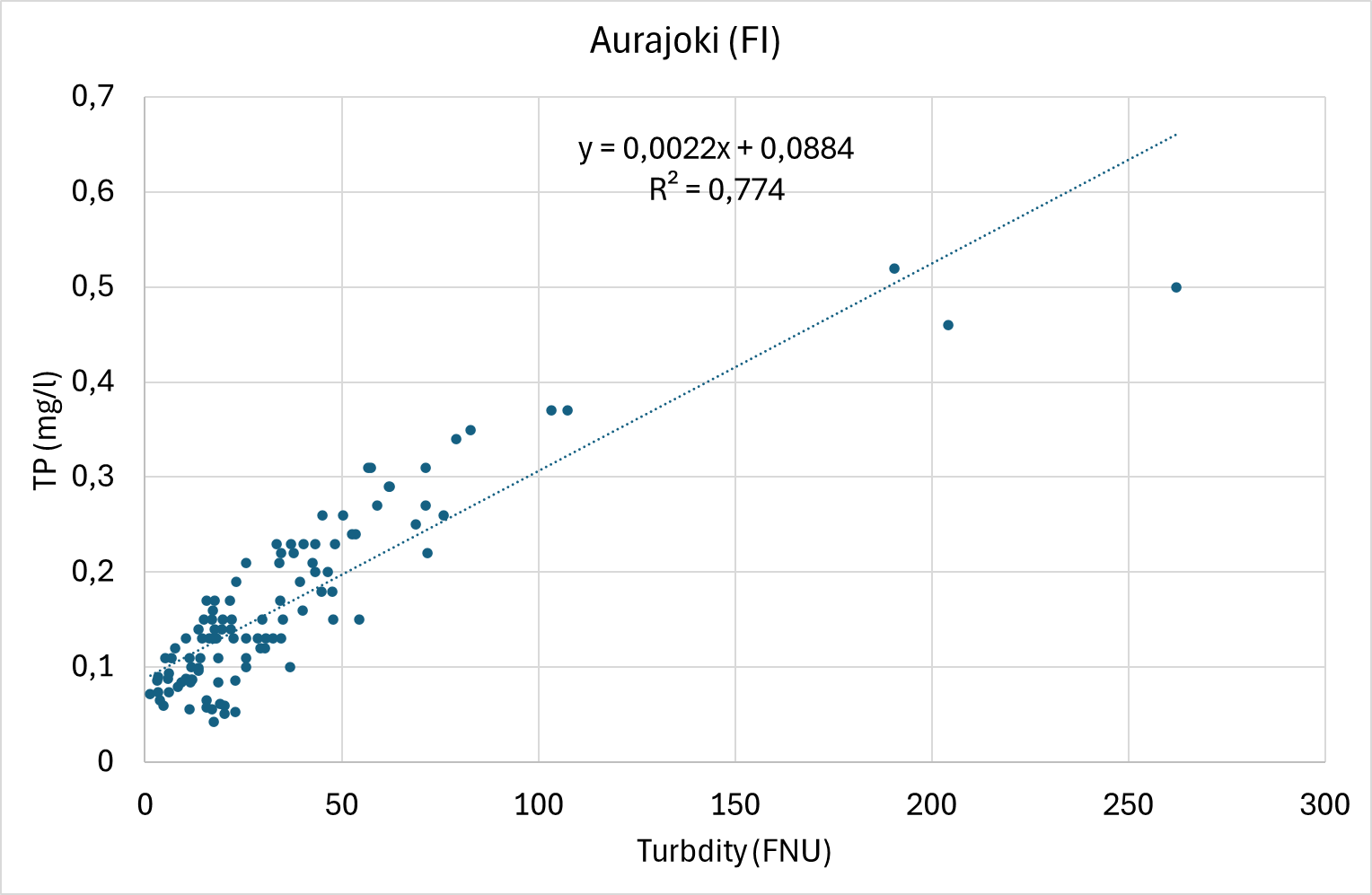 | 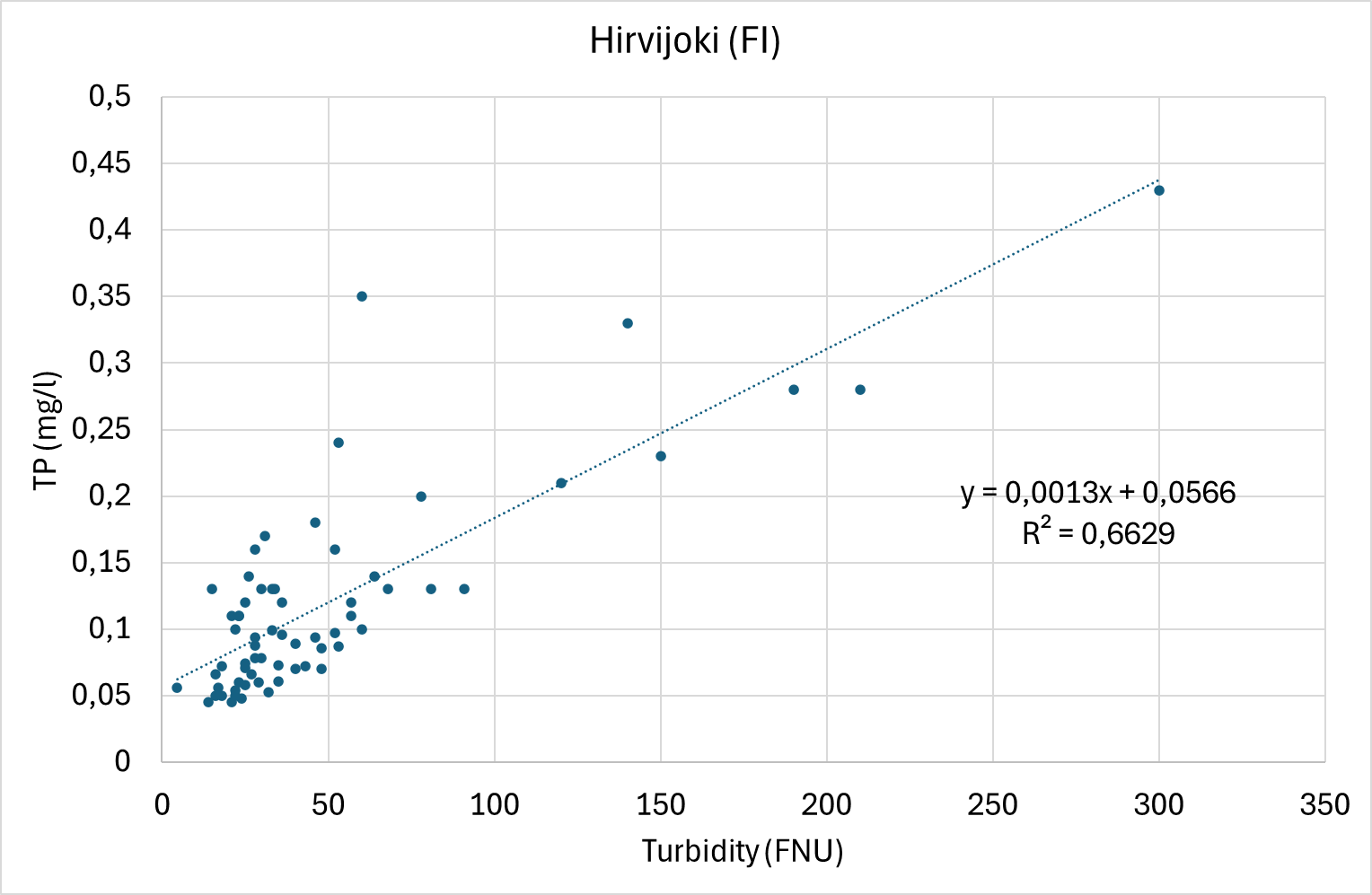 |
| 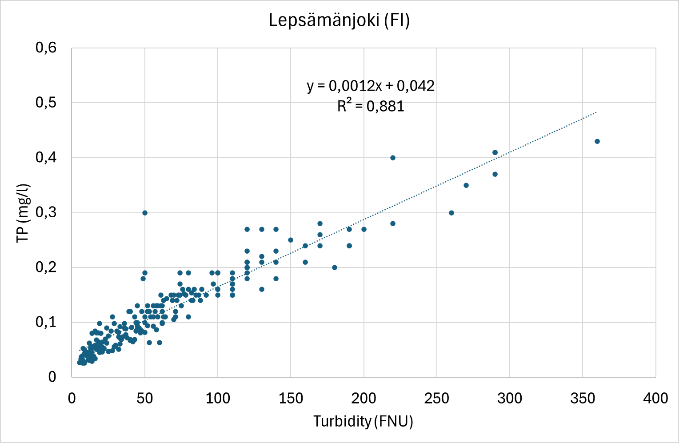 | 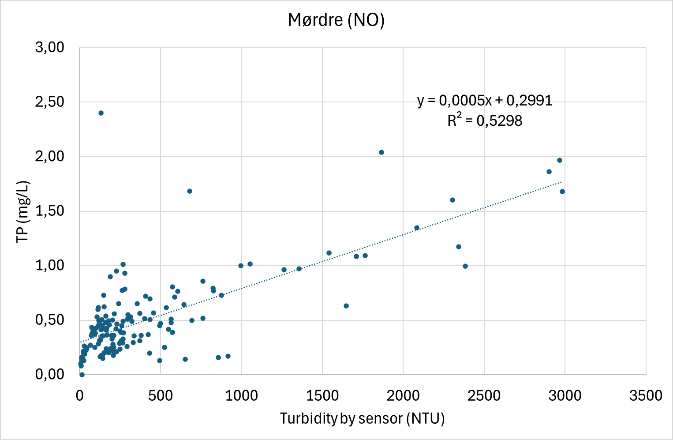 |
| 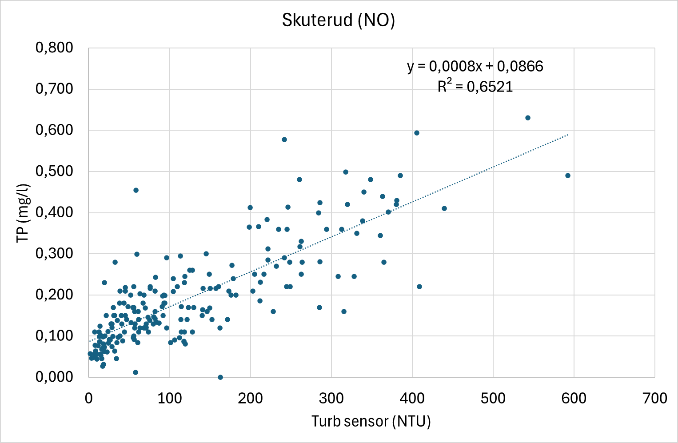 | 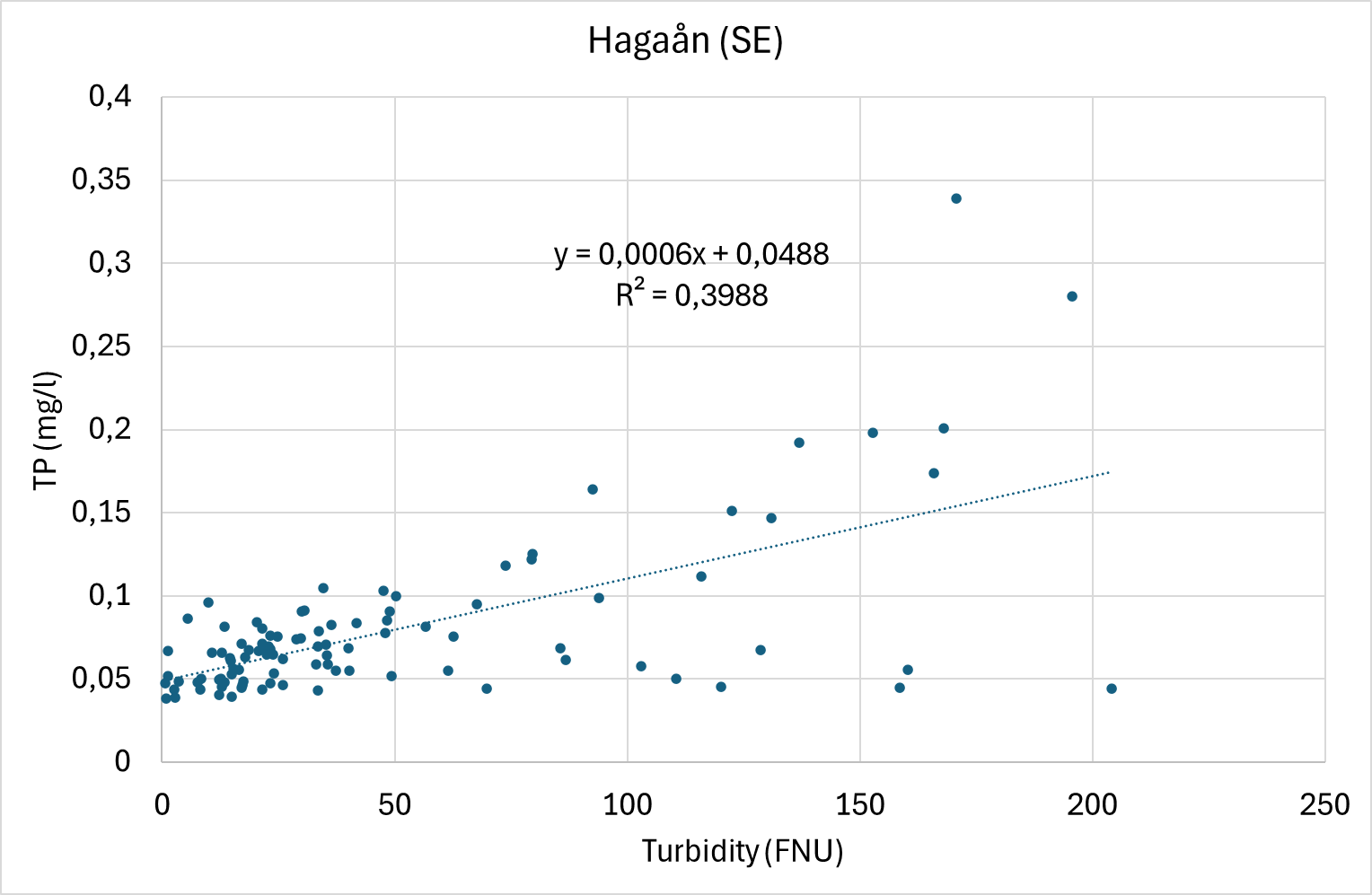 |
| 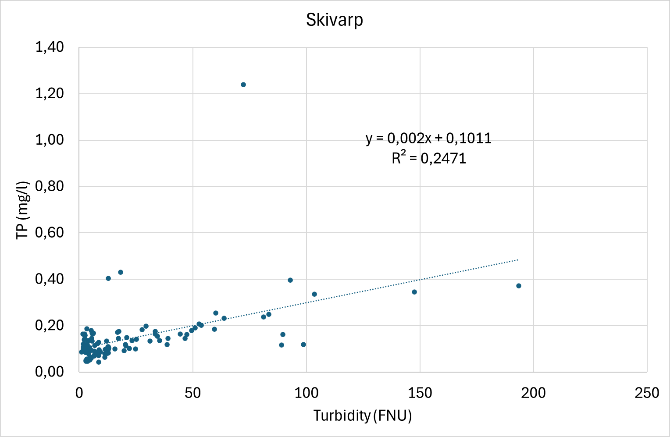 | 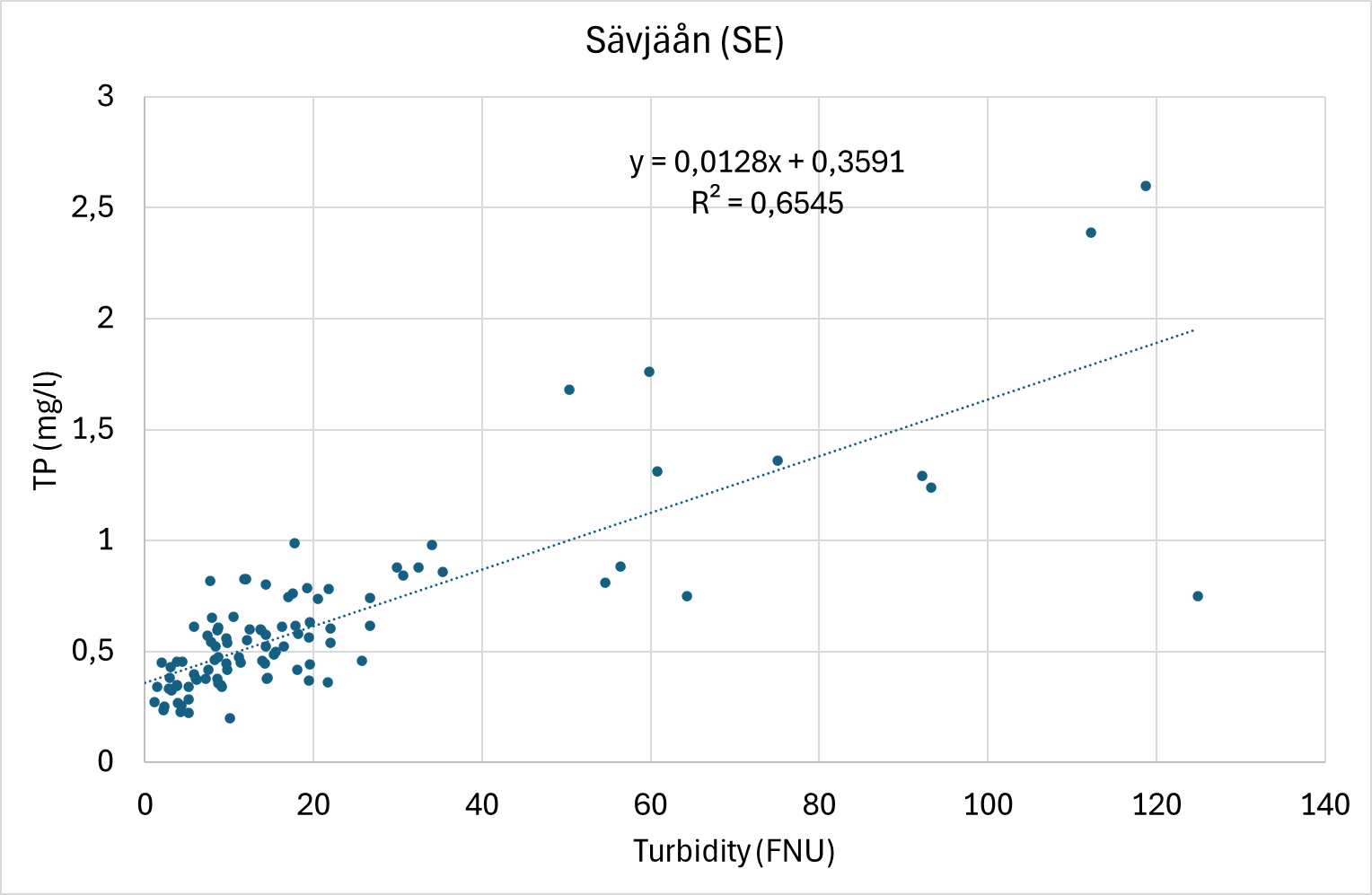 |

**Figure S1.** Calibrations, equations and R^2^s between turbidity by sensor (x-axis) and TP-concentrations from grab samples (y-axis) for the 10 streams.


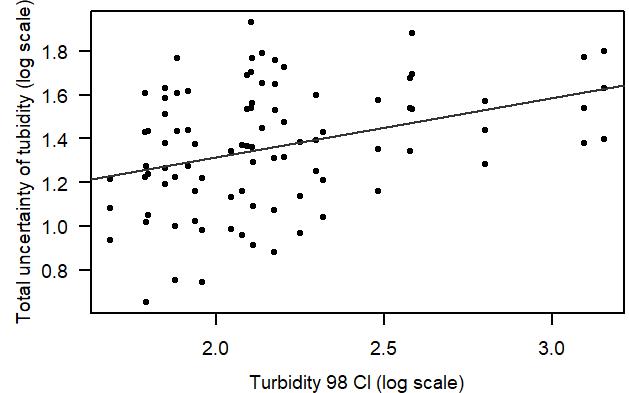

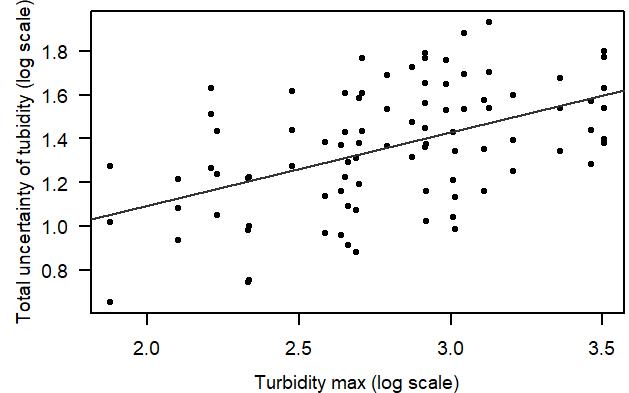

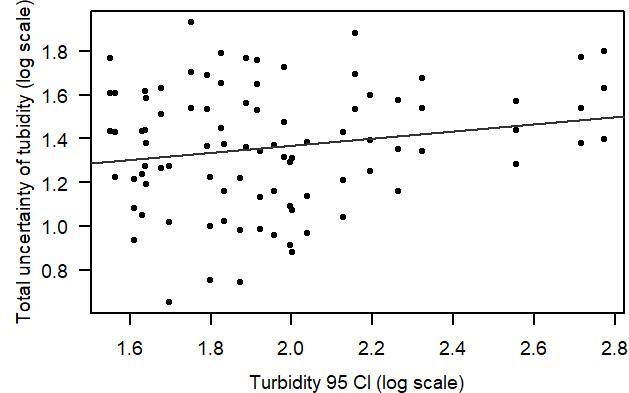

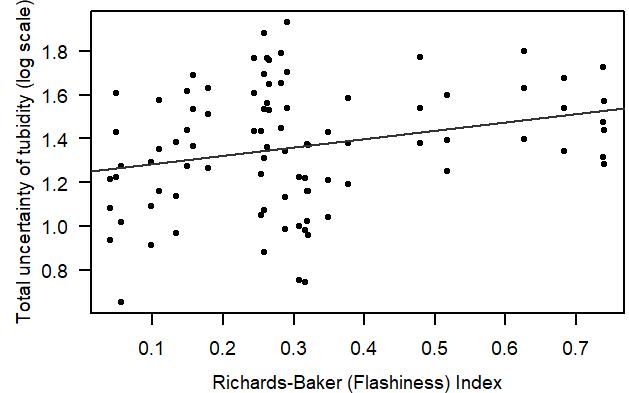

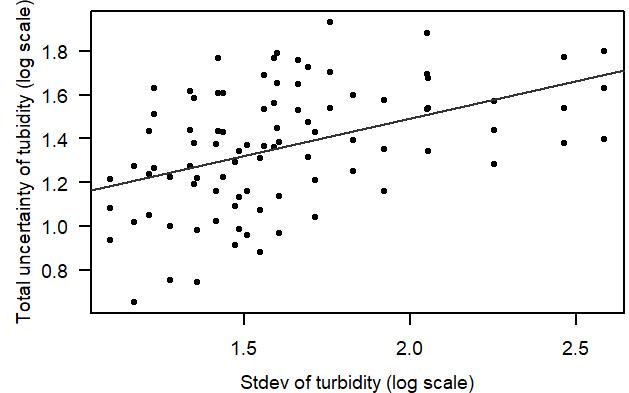

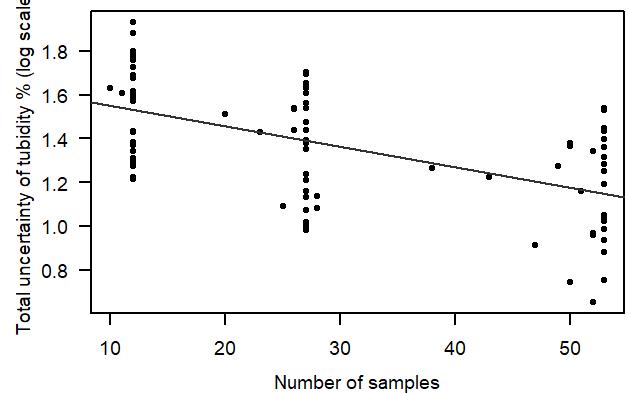

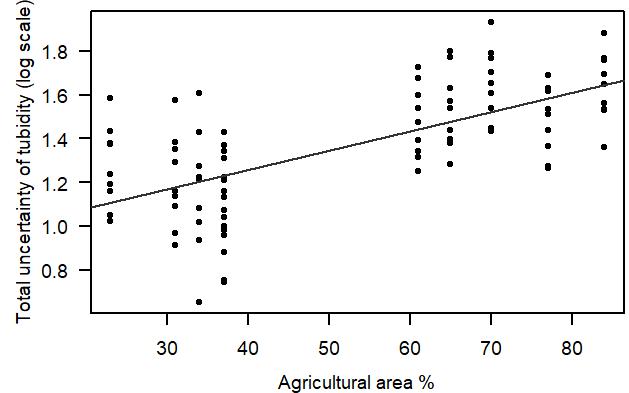

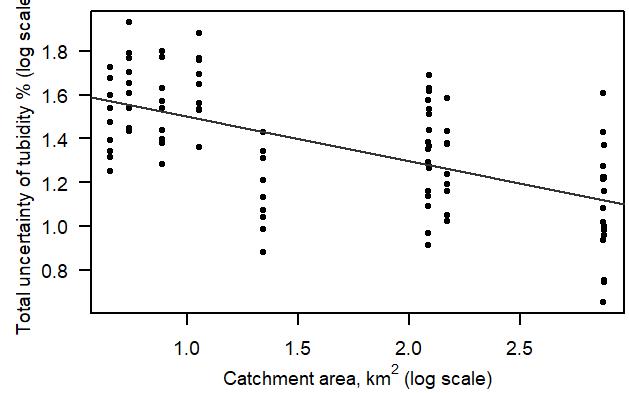

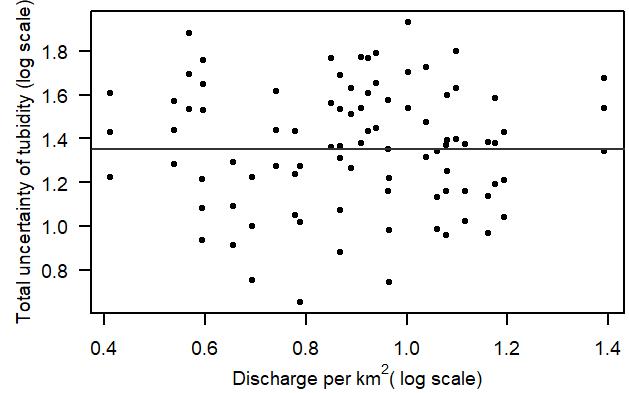

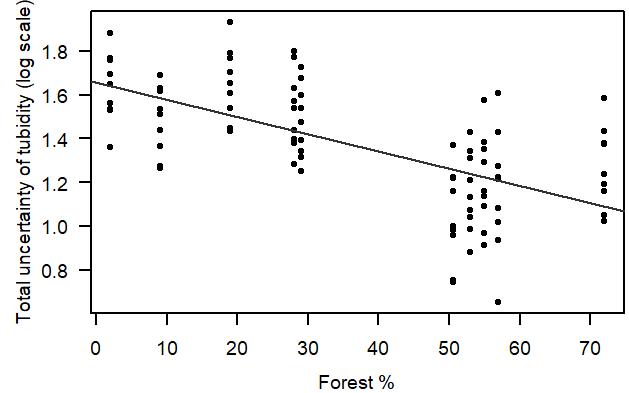

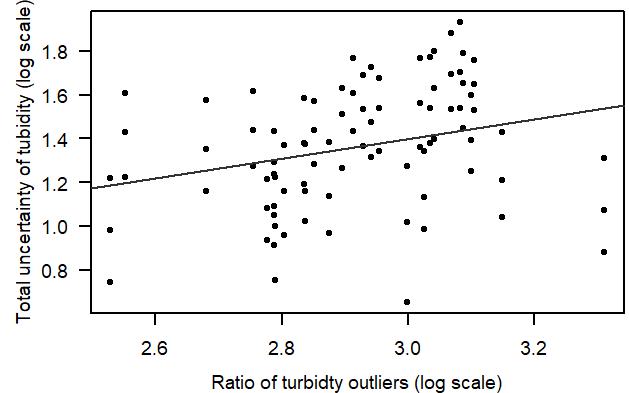


**Figure S2.** Calibrations between total uncertainty and tested possible explanatory factors.


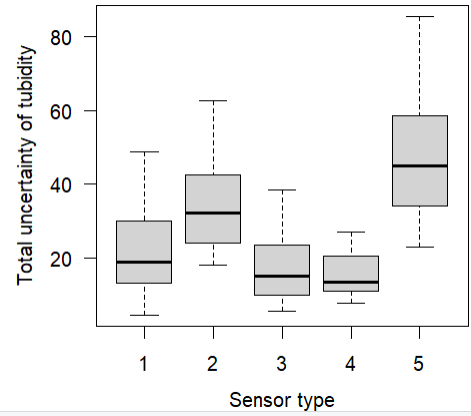


**Figure S3** Total uncertainty of turbidity (%) shown for each sensor type: 1: Swedish; 2: Norwegian; 3 and 4: Finnish, where 4 is for the Lepsämänjoki stream; and 5: Danish sensor type.

A Kruskal–Wallis test showed a significant difference in *Uncertainty* across the five levels, where Dunn’s post‑hoc test with Holm correction revealed that groups **1 and 2**, **1 and 5**, **2 and 3**, **2 and 4**, **3 and 5**, and **4 and 5** differed significantly (adjusted p < .05). No significant differences were observed between **1 and 3**, **1 and 4**, **2 and 5**, or **3 and 4**.
